# Transcriptome analysis reveals pituitary lncRNA, circRNA and mRNA affecting fertility in high- and low-yielding goats

**DOI:** 10.3389/fgene.2023.1303031

**Published:** 2023-12-12

**Authors:** Shuaixiang Mao, Shucan Dong, Biwei Hou, Yaokun Li, Baoli Sun, Yongqing Guo, Ming Deng, Dewu Liu, Guangbin Liu

**Affiliations:** College of Animal Science, South China Agricultural University, Guangzhou, China

**Keywords:** Leizhou goat, pituitary, fertility, lncRNA, circRNA, mRNA

## Abstract

The pituitary gland serves as the central endocrine regulator of growth, reproduction, and metabolism and plays a crucial role in the reproductive process of female animals. Transcriptome analysis was conducted using pituitary gland samples from Leizhou goats with varying levels of fecundity to investigate the effects of long noncoding RNA (lncRNA), circular RNA (circRNA), and mRNA regulation on pituitary hormone secretion and its association with goat fecundity. The analysis aimed to identify lncRNAs, circRNAs, and mRNAs that influence the fertility of Leizhou goats. GO and KEGG enrichment analyses were performed on differentially expressed lncRNAs, circRNAs, and mRNAs and revealed considerable enrichment in pathways, such as regulation of hormone secretion, germ cell development, and gonadotropin-releasing hormone secretion. The pituitary lncRNAs (ENSCHIT00000010293, ENSCHIT00000010304, ENSCHIT00000010306, ENSCHIT00000010290, ENSCHIT00000010298, ENSCHIT00000006769, ENSCHIT00000006767, ENSCHIT00000006921, and ENSCHIT00000001330) and circRNAs (chicirc_029285, chicirc_026618, chicirc_129655, chicirc_018248, chicirc_122554, chicirc_087101, and chicirc_078945) identified as differentially expressed regulated hormone secretion in the pituitary through their respective host genes. Additionally, differential mRNAs (GABBR2, SYCP1, HNF4A, CBLN1, and CDKN1A) influenced goat fecundity by affecting hormone secretion in the pituitary gland. These findings contribute to the understanding of the molecular mechanisms underlying pituitary regulation of fecundity in Leizhou goats.

## 1 Introduction

Goats are among the earliest domesticated animals and widely distributed worldwide as livestock. Given their small size and high productivity, they have no competition with humans in terms of food, and they can provide human beings with meat, milk, and other daily necessities (Ahlawat et al., 2015; Yao et al., 2023). They also play a crucial role in animal husbandry. With the rapid development of the social economy and the improvement of peoples living standards, meat consumption has increased substantially. However, the slow development of the mutton goat industry has led to a widening gap between production and consumption (Kantono et al., 2021; Li et al., 2022). Genetic enhancement of the reproductive rate in low-yield animals can help fill this production–demand gap (Islam et al., 2020). Litter size is a crucial economic indicator in the goat industry. However, goats generally have a low reproductive rate, with an average litter size of 1–2, and their low fecundity greatly inhibits the development of the goat breeding industry (Wang et al., 2012). Leizhou goats, a superior goat breed in southern China, are known for their early maturity, fast growth, and high-quality meat (Dong et al., 2023). However, a difference in fertility exists between low- and high-fertility populations of Leizhou goats. The use of high-throughput sequencing technology to identify genes associated with reproductive traits in Leizhou goats can provide valuable insights into the genetic factors that influence goat fertility at the molecular level.

The litter size of goats is closely related to the number of mature oocytes released during the ovulation cycle (Zhao et al., 2015). Follicle development and maturation are regulated by the secretion of various hormones from the hypothalamic–pituitary–gonadal axis and by several growth factors and cytokines expressed by the ovaries and follicles (Chronowska, 2014). The pituitary gland is a key regulator of the hypothalamic–pituitary–gonadal axis (Wan et al., 2022b) and the central endocrine regulator of growth, reproduction, and metabolism. The secretion of synthetic hormones in adenohypophysis is not only regulated by gonadotropin-releasing hormones (GnRHs; Li et al., 2019), but also influenced by certain noncoding RNAs present in pituitary cells (Wan et al., 2022a). However, research on the underlying molecular regulatory mechanisms is limited.

The regulation of goat reproduction is a complex biological process that involves coordinated gene interactions. The pituitary gland plays an essential role in regulating reproductive performance, and this regulation involves coding and noncoding RNAs. Studies on the effects of long noncoding and circular RNAs on reproductive performance have focused on the ovaries (Miao et al., 2017; Liu et al., 2021; Zhang et al., 2022) and granulosa cells (Li et al., 2018; Li X. et al., 2021; Zhao et al., 2023), and research on their effects on reproductive performance in the pituitary gland is limited (Yang et al., 2020). Long noncoding RNA (lncRNA) is transcribed by RNA polymerase II/III and is a type of RNA molecule whose length exceeds 200 bp. It is widely present in eukaryotes but cannot encode functional proteins (Zhang et al., 2020; Lee and Kang, 2022). Circular RNA (circRNA) is an endogenous, single-stranded, covalently closed noncoding RNA. Its unique circular structure renders circRNA more stable than linear RNA (Du Toit, 2013; Gao et al., 2023). Noncoding RNAs influence the expression of coding genes through competitive regulatory networks, thereby affecting various biological processes (Qiannan et al., 2020). Transcriptome sequencing technology provides information on nearly all coding and noncoding genes, thus enabling us to explore the mutual regulatory relationships between genes and elucidate the underlying molecular mechanisms (Costa et al., 2010). Therefore, transcriptome technology can help in understanding the molecular mechanisms of pituitary regulation of animal reproduction by identifying genes associated with pituitary regulation of reproductive performance.

This study utilized transcriptome technology to investigate the pituitary glands of low- and high-fertility Leizhou goats. The objective was to identify differentially expressed functional genes that may affect the reproductive performance of Leizhou goats. The findings can serve as a reference for understanding the molecular mechanisms that regulate the reproduction of Leizhou goats and provide a theoretical foundation for studying the prolific traits of this breed.

## 2 Materials and methods

### 2.1 Experimental animals and sample collection

Seven healthy Leizhou female goats aged between 3.5 and 4.5 years were selected for this study. All the goats had more than three previous litters and were managed under consistent-feeding and management conditions. The goats were divided into two groups on the basis of fertility: high-fertility group (*n* = 3) where ewes had two or more litters per pregnancy and low-fertility group (*n* = 4) where ewes had only one litter per pregnancy (Supplementary Table S11). After synchronized estrus, all seven Leizhou goats were slaughtered. The pituitary glands were collected, immediately placed in frozen tubes, flash-frozen using liquid nitrogen, and stored for the long term at −80°C in a refrigerator.

### 2.2 RNA extraction, cDNA library preparation, and sequencing

Total RNA was extracted from the samples by using the Trizol reagent (Thermo Fisher, Shanghai, China) in accordance with the manufacturer’s instructions, and ribosomal RNA in total RAN was removed with the Ribo-Zero rRNA Removal Kit (Illumina, Inc.). The absence of any genomic DNA contamination was confirmed by 1% agarose gel electrophoresis for RNA degradation. The total amount of RNA and its integrity were determined using the Agilent 2100 Bioanalyzer and RNA 6000 Nano LabChip Kit (Agilent, Santa Clara, United States). The samples that met the test requirements were sent to Shanghai Personalbio Technology Co., Ltd. (Shanghai, China). Sequencing was performed using the Illumina HiSeq 2500 platform. Exactly 1 μL of total RNA from each sample was used for library construction, and the RNA was cleaved into 200- to 300-bp fragments, followed by first-strand cDNA synthesis using random hexamer primers and reverse transcriptase and second-strand cDNA synthesis. Double-end sequencing (150 bp) was performed with the NEB Next Ultuar Directional RNA Library Prep Kit for Illumina (NEB, Ispawich, United States).

### 2.3 Quality assessment of raw sequencing data and assembly of transcripts

After the raw image data generated by the HiSeq platform were converted into raw data in FASTQ format, the raw sequence data were quality checked using Cutadapt to remove joints and low-quality reads. The filtered clean reads were aligned to the *Capra hircus* reference genome by using HiSAT2. After the comparison bam file was obtained, String Tie software was used to align the reads on the genome, count the expression levels of transcripts in each sample, and standardize lncRNA and mRNA as transcripts per kilobase million (FPKM) and circRNA as transcripts per million (TPM).

### 2.4 Screening of lncRNAs and circRNAs

In the transcript assembly results, single-exon transcripts with low expression were filtered out. Transcripts with an exon number greater than or equal to 2 and a length of at least 200 bp were selected. Cuffcompare software was used to screen the transcripts with overlapping exon regions of the database annotation, and the lncRNA in the database with overlapping exon regions of this spliced transcript was included in the subsequent analysis as the database annotation lncRNA. The transcripts without coding ability judged by PLEK, CNCI, and Pfamscan software were high-confidence lncRNAs. The expression level of lncRNA in each sample was determined and homogenized by FPKM. After alignment with the reference genome, the nonalignment reads were used to identify circRNAs. After alignment of the anchor sequence of each sample with the reference genome, we combined the alignment results of all the samples and used find_circ to identify circRNAs. Then, the high-confidence circRNAs were filtered based on the following criteria: 1) breakpoints = 1; 2) anchor_overlap ≤ 2; 3) edit ≤ 2; 4) n_uniq > 2, n_uniq > samples, and n_uniq > int (1/2 samples); 5) best_qual_A > 35 or best_qual_B > 35; and 6) circRNA < 100 k in length. The expression level of circRNAs was estimated by TPM.

### 2.5 Differential expression and enrichment analyses

Differential analysis of gene expression in the two groups was performed using the R language DEseq package, and genes that met the differential fold |log2 Fold Change| > 1 and *p* < 0.05 were screened as differentially expressed genes. Gene ontology (GO) enrichment analysis was performed using top GO. The significantly enriched GO term was determined by the hypergeometric distribution method (the criterion for significant enrichment was *p* < 0.05), and KEGG pathway enrichment analysis was performed using the Cluster Profiler software for significant enrichment (the criterion for significant enrichment was *p* < 0.05). On the basis of the GO and KEGG enrichment analysis results and biological significance, the target genes were selected for follow-up study.

### 2.6 LncRNA–mRNA network co-expression construction

Cis-target gene regulation of lncRNAs depends on their nearby protein-coding genes, which are usually considered to be their target genes with 100 kb located upstream and downstream. The Pearson correlation test was performed to calculate the correlation coefficients of pituitary DE lncRNAs and DE mRNAs, with Spearman correlation coefficient > 0.8 and *p* < 0.05 as a conditional screen. The results were visualized using Cystoscape software (v3.9.1).

### 2.7 Construction of protein–protein interaction network

The interaction between predicted mRNA translation proteins was predicted using the STRING database with a confidence score ≥ 0.4. Protein–protein interaction (PPI) network regulation was visualized by Cytoscape (v3.9.1).

### 2.8 RNA-seq validation by quantitative real-time PCR

In accordance with the sequencing results, three lncRNA, circRNA, and mRNA were selected for expression verification. GAPDH was used as the reference gene of lncRNA, circRNA, and mRNA, and reverse transcription was performed using a Takara reverse transcription kit. The 2 × Ultra SYBR Green qPCR Mix fluorescence quantitative kit was employed to detect the gene expression. The real-time quantitative PCR (RT-qPCR) cycling parameters were as follows: predenaturation at 95°C for 10 min, followed by 40 cycles of 5 s at 95°C and 60°C for 20 s. Three biological replicates were used for each assay. The relative expression of the target genes was analyzed using the 2^−ΔΔCT^ method. The primer sequences are shown in Table 1.

**TABLE 1:** Primer sequences for RT-qPCR.

Name	Primer type	Primer sequence
MSTRG.52870.6	Forward	ACA​ACA​GTG​TCA​GCA​TCC​GT
	Reverse	TTG​GAG​TCC​CAT​TAT​GGC​GG
MSTRG.33887.2	Forward	TGT​TTC​CTC​GAC​GTG​ACC​TG
	Reverse	AAG​ACG​ACT​TTG​GGC​TGG​AG
ENSCHIT00000008199	Forward	TGT​TGC​TTC​CTG​ACC​TGC​AT
	Reverse	GCC​GCA​CCA​TCT​ATT​TCT​GC
chicirc_061339	Forward	GAG​AAA​TCT​CAG​CAG​GCG​GT
	Reverse	GTT​CCA​TGG​GCT​TTT​GGC​AG
chicirc_002310	Forward	CGT​TGT​CAC​GAT​CAC​GCA​TC
	Reverse	AGC​CTC​GAA​ATC​CAG​CAC​AA
chicirc_131778	Forward	TGG​TGA​CGT​GGA​AAA​GAC​CC
	Reverse	CTC​CTC​GTC​CGT​GGT​GTT​C
CDKN1A	Forward	CCA​GAC​CAG​CAT​GAC​AGA​TTT​C
	Reverse	GTG​ACA​GCA​AGC​AGC​GTA​TG
SYCP1	Forward	ATC​TGC​GTA​CAC​CTG​CCA​AA
	Reverse	TCC​TCT​GAA​ACC​ATG​CTC​AAA
RIMS4	Forward	GGA​GTT​TGT​CTG​GCA​TCG​GA
	Reverse	CCT​TGA​TGT​AGG​CCG​CTG​G

## 3 Results

### 3.1 Quality control of RNA-seq sequencing data

Prior to further analysis, quality control analyses were performed on raw reads from the low- and high-fertility groups. Seven independent cDNA libraries were constructed from pituitary tissue RNA of the low-fertility group (LZ_L) and high-fertility group (LZ_H). The sequencing profiles are shown in Table 2. A total of 734,700,378 raw reads were generated from the seven sequencing libraries. After quality control, 732,086,222 clean reads were left. The number of bases with 99.9% or higher accuracy per sample was 92.48%–93.46% of the total number of bases. Clean data were aligned to the reference genome, and over 84.1% of the reads were accurately aligned with a high matching rate. About 1.78%–3.88% of these clean reads had multiple aligned positions, and 96.12%–98.22% of them had single aligned positions. These data show that the sequencing results were of high quality and could be used for the subsequent analysis.

**TABLE 2:** Pituitary RNA sequencing profiles of low- and high-fertility Leizhou goats.

Sample	LZ_L1	LZ_L2	LZ_L3	LZ_L4	LZ_H1	LZ_H2	LZ_H3
Raw reads	105,065,052	103,763,540	106,273,534	102,644,634	106,205,704	106,878,816	103,869,098
Clean reads	104,649,542	103,389,924	105,891,248	102,277,412	105,838,156	106,542,068	103,497,872
Clean reads (%)	99.6	99.63	99.64	99.64	99.65	99.68	99.64
Q30 (%)	93.02	93.31	93.46	93	92.92	93.32	92.48
Q20 (%)	97.13	97.26	97.32	97.16	97.13	97.3	96.93
Total_Mapped (%)	84.42	88.02	87.43	85.34	86.96	87.62	84.1
Multiple_Mapped (%)	1.78	2.18	3.88	1.83	1.95	3.61	2.7
Uniquely_Mapped (%)	98.22	97.82	96.12	98.17	98.05	96.39	97.3

### 3.2 Differential expression analysis of lncRNA, circRNA, and mRNA

**FIGURE 1:**
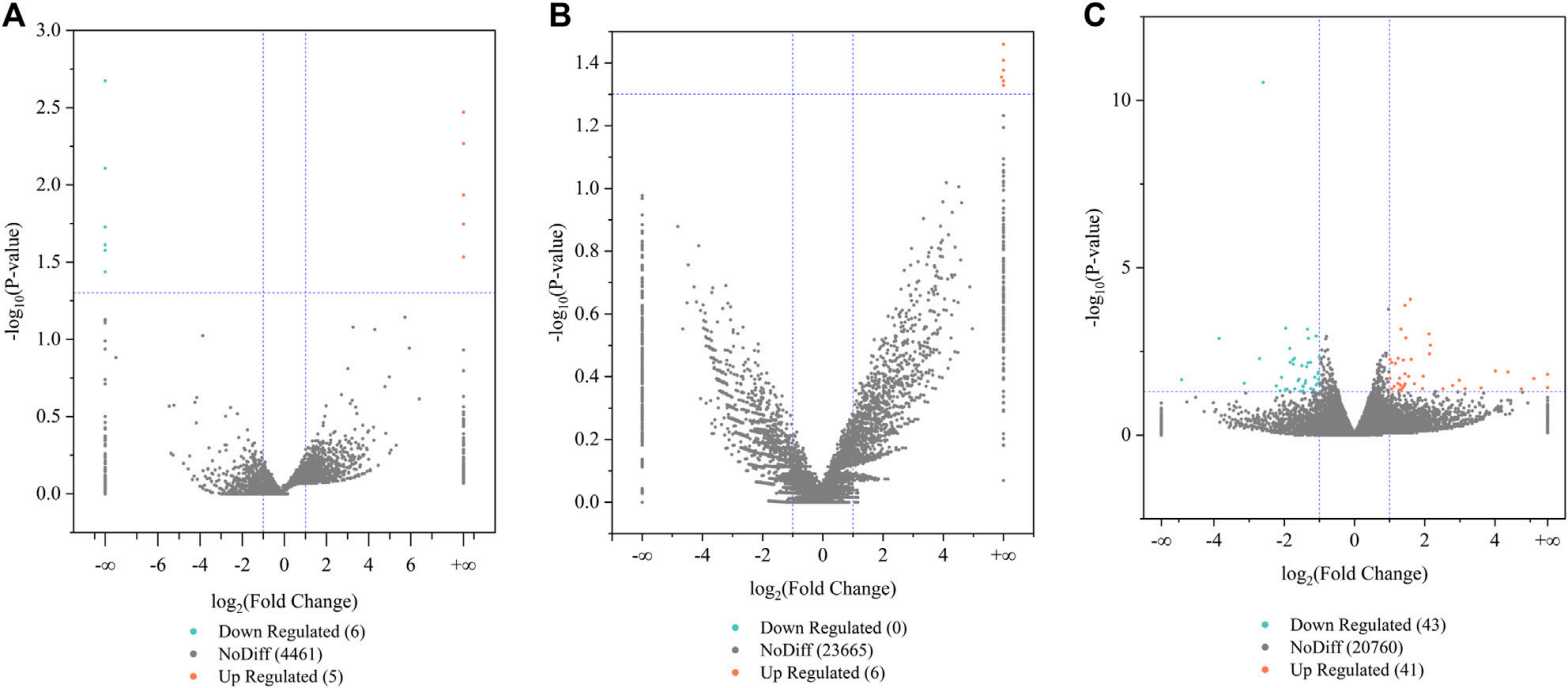
Analysis of differentially expressed lncRNA, circRNA, and mRNA. **(A)** Volcano plots of DE lncRNAs, **(B)** volcano plots of DE circRNAs, and **(C)** volcano plots of DE mRNA. Orange and green indicate upregulated and downregulated expression levels, respectively.

Differences in the gene or transcript expression of the pituitary, as the central regulator of the goat hypothalamic–pituitary–ovarian axis, may affect the reproductive ability of Leizhou goats. We screened for DE lncRNAs, DE circRNAs, and DE mRNAs between the low- and high-fertility groups on the basis of |log2 Fold Change| > 1 and *p* < 0.05. The low-fertility pituitary samples were used as the control group, and a total of 4,472 lncRNAs were detected in the low- and high-fertility group pituitary samples. Among these lncRNAs, 11 differentially expressed lncRNAs were identified, including 5 upregulated lncRNAs and 6 downregulated lncRNAs. The five upregulated lncRNAs mainly included MSTRG.52870.6, ENSCHIT00000005480, and MSTRG.45842.5, and the six downregulated lncRNAs mainly included ENSCHIT00000009877, ENSCHIT00000009854, and ENSCHIT00000008199 (Supplementary Table S1; Figure 1A).

The volcano plot revealed the identification of 23,671 circRNAs in the two groups. Six differentially expressed circRNAs were present compared with the low-fertility group, all of which were upregulated genes and mainly included chicirc_061339, chicirc_002310, and chicirc_131778 (Supplementary Table S1; Figure 1B).

The volcano plot results also revealed the identification of 20,844 mRNAs in the two groups. A total of 84 mRNAs were differentially expressed, 41 mRNAs were upregulated, and 43 mRNAs were downregulated compared with the low-fertility group of Leizhou goats. The upregulated mRNAs mainly included RIMS4, SYCP1, LMNTD2, and NAP1L2, and the downregulated mRNAs mainly included SERPINE1, IL33, SLC44A5, and BAZ1A (Supplementary Table S1; Figure 1C). These differentially expressed transcripts are valuable for further studies on the reproductive performance of Leizhou goats.

### 3.3 Enrichment analysis of the pituitary function

#### 3.3.1 Functional enrichment analysis of the target genes of differentially expressed lncRNAs

**FIGURE 2:**
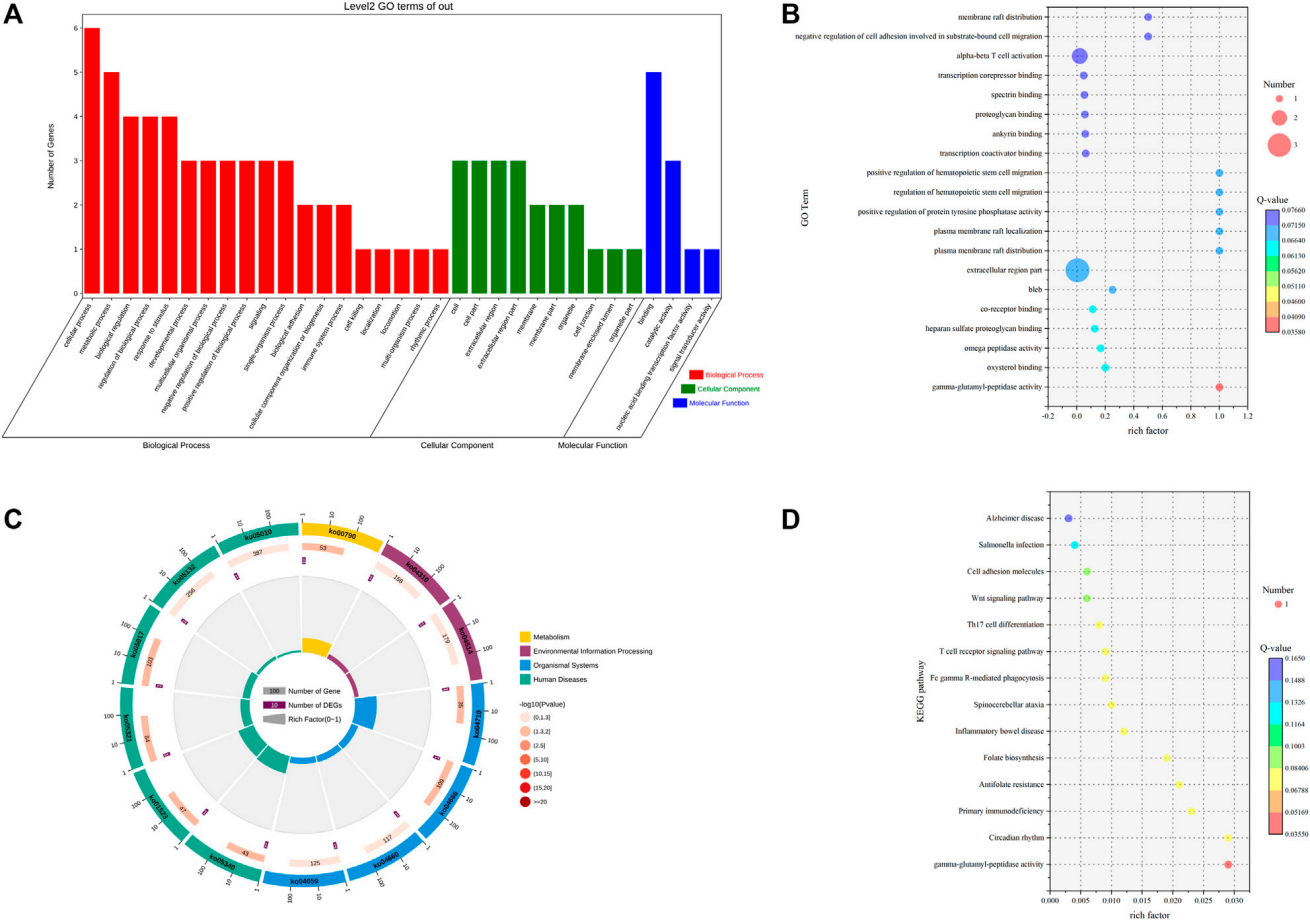
GO and KEGG analyses of DE lncRNA target genes. **(A)** GO term of genes in BP, CC, and MFs. **(B)** GO enrichment bubble map of DE lncRNA target genes. **(C)** Enrichment circle diagram of the KEGG pathway of DE lncRNA target genes. **(D)** Bubble diagram of KEGG enrichment of DE lncRNA target genes.

To thoroughly understand the biological functions of differentially expressed lncRNAs in the pituitary of low- and high-fertility groups, we predicted potential targets in terms of cis-regulatory relationships. We searched for 100-kb protein-coding genes upstream and downstream of the differentially expressed lncRNAs and performed GO function enrichment analysis on the target genes of the 11 differentially expressed lncRNAs. We found that the target genes of the differentially expressed lncRNAs were enriched to 853 GO terms. Differential significant enrichment analysis of the enrichment results yielded a total of 306 GO terms (*p* < 0.05) that were significantly enriched, of which 275 GO terms were for biological processes, 8 were for cellular composition, and 23 were for molecular functions. The four biological processes with the highest gene enrichment were cellular, metabolic, biological regulation, and regulation of the biological process, and the four cellular components were cell, cell part, extracellular region part, and extracellular region. The four most enriched molecular functions were nucleic acid binding transcription factor activity, binding, signal transducer factor activity and catalytic activity (Supplementary Table S2; Figures 2A, B).

KEGG enrichment analysis of the 11 differentially expressed lncRNA target genes resulted in 13 pathways, with seven significantly enriched KEGG pathways (*p* < 0.05), including folate biosynthesis, spinocerebellar ataxia, Fc gamma R-mediated phagocytosis, and Wnt signaling pathway (Supplementary Table S3; Figures 2C, D).

#### 3.3.2 Functional enrichment analysis of host genes of differentially expressed circRNAs

**FIGURE 3:**
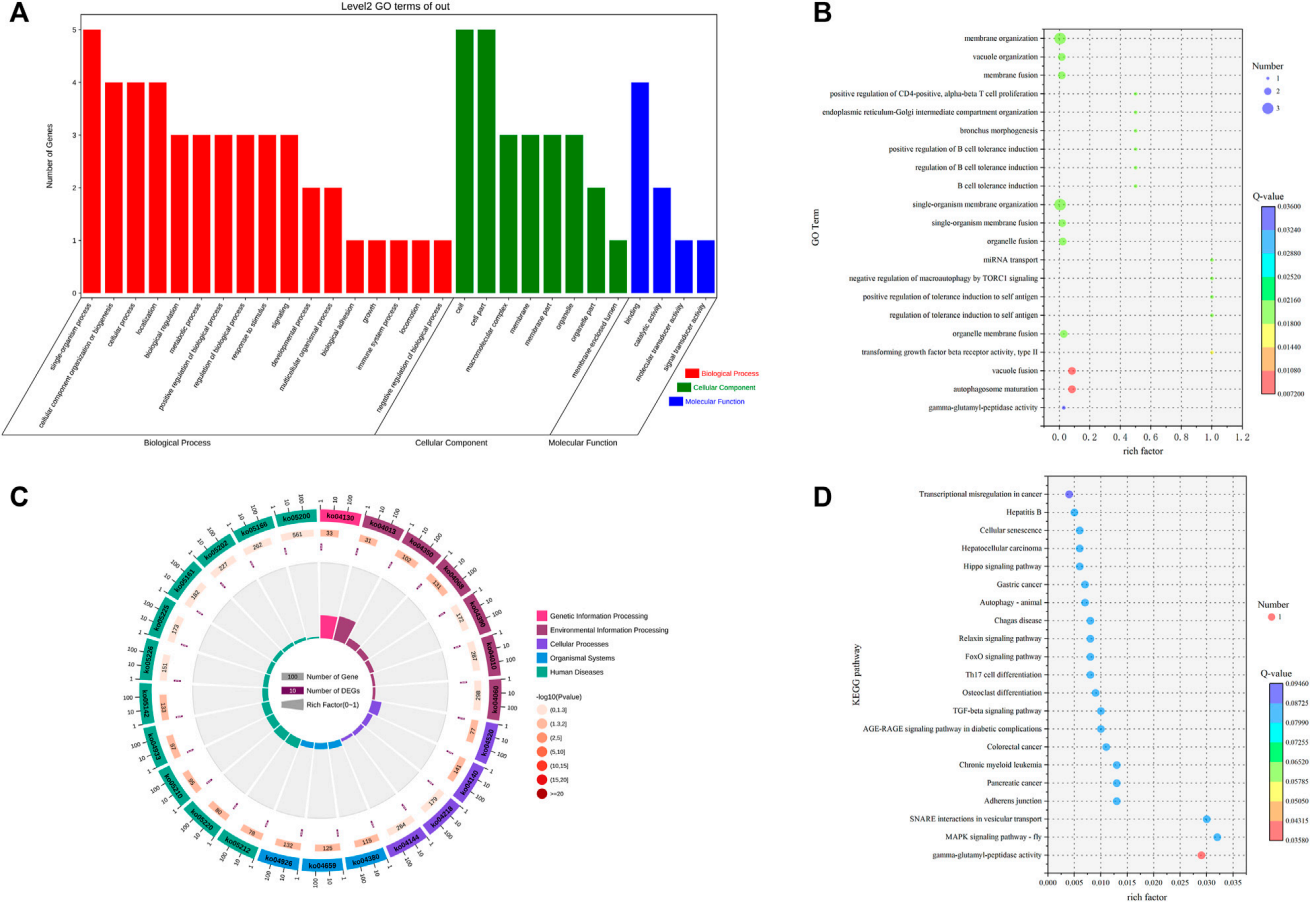
GO and KEGG analyses of DE circRNA host genes. **(A)** GO term of genes in BP, CC, and MFs. **(B)** GO enrichment bubble map of DE circRNA host genes. **(C)** Enrichment circle diagram of the KEGG pathway of DE circRNA host genes. **(D)** Bubble diagram of KEGG enrichment of DE circRNA host genes.

To explore the functions of differential circRNAs in the pituitary of Leizhou goats with high and low fertility, we performed GO functional enrichment analysis on the host genes of the six differentially expressed cirRNAs and found that the host genes of the differentially expressed circRNAs were enriched to 693 GO terms. Differential significant enrichment analysis was performed on the enrichment results. A total of 322 GO terms (*p* < 0.05) were significantly enriched, of which 268 GO terms were for biological processes, 19 were for cellular components, and 35 were for molecular functions. The four biological processes with the highest gene enrichment were single-organism process, cellular component organization or biogenesis, cellular process, and localization, and the four cellular components with the highest gene enrichment were cell, cell part, macromolecular complex, and membrane. The four most enriched molecular functions were molecular transducer activity, binding, signal transducer activity and catalytic activity (Supplementary Table S4; Figures 3A, B).

KEGG enrichment analysis was performed on the host genes of six differentially expressed circRNAs. A total of 25 pathways were enriched, of which 14 were significantly enriched (*p* < 0.05). These pathways included the MAPK signaling pathway-fly, TGF-beta signaling pathway, FoxO signaling pathway, and autophagy-animal (Supplementary Table S5; Figures 3C, D).

#### 3.3.3 Functional enrichment analysis of differentially expressed mRNAs

**FIGURE 4:**
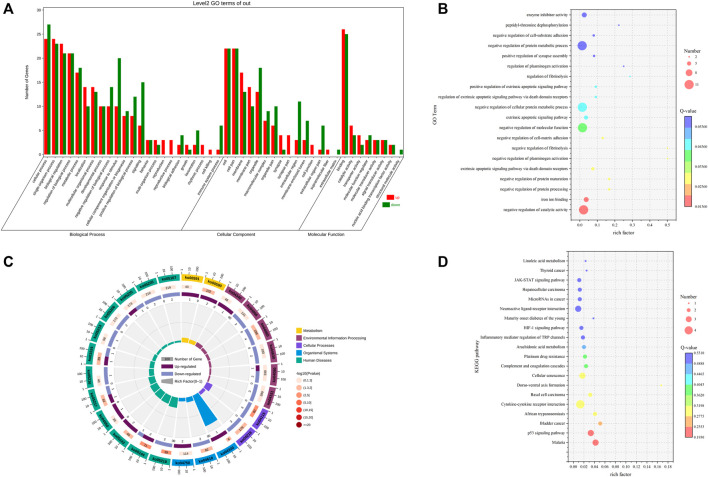
GO and KEGG analyses of DE mRNA. **(A)** GO term of genes in BP, CC, and MFs. **(B)** GO enrichment bubble map of DE MrnA. **(C)** Enrichment circle diagram of the KEGG pathway of DE mRNA. **(D)** Bubble diagram of KEGG enrichment of DE mRNA.

GO functional enrichment analysis was performed on the 84 differentially expressed mRNAs to predict the potential biological function of differential mRNAs, and the results showed that the differentially expressed mRNAs were enriched to 2,187 GO terms. The differential significant enrichment analysis of theenrichment results revealed that a total of 502 GO terms (*p* < 0.05) were significantly enriched, of which 412 GO terms were for biological processes, 35 were for cellular components, and 55 were for molecular functions. The four biological processes with the highest gene enrichment were single-organism process, biological regulation, cellular process, and regulation of the biological process, and the four cellular components with the highest gene enrichment were cell, cell part, membrane part, and membrane. The four most enriched molecular functions were molecular function regulator, binding, signal transducer activity, and catalytic activity (Supplementary Table S6; Figures 4A, B). The genes enriched to reproduction-related genes were GABBR2, SYCP1, HNF4A, CBLN1, and CDKN1. These genes may play an important role in the regulation of reproduction in Leizhou goats.

KEGG enrichment analysis was performed on the 84 differentially expressed mRNAs to further clarify the contribution of specific signaling pathways to goat fecundity, and the results showed that a total of 101 pathways were enriched, including 11 significantly enriched KEGG pathways (*p* < 0.05). These pathways included the p53 signaling pathway, cytokine–cytokine receptor interaction, cellular senescence, and arachidonic acid metabolism These signaling pathways may play a role in reproduction and require further research (Supplementary Table S7; Figures 4C, D).

### 3.4 Construction of lncRNA–mRNA and circRNA–mRNA regulatory networks

**FIGURE 5:**
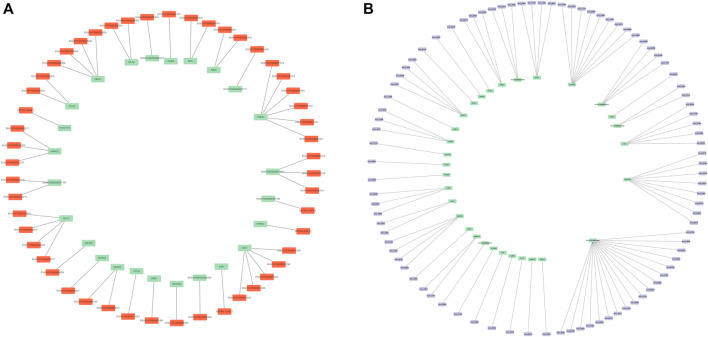
**(A)** DE lncRNA–mRNA network mapping. **(B)** DE circRNA–mRNA network mapping. Orange represents DE lncRNA, and purple represents DE circRNA. The green color in **(A)** represents the target gene, and the green color in **(B)** represents the host gene.

To further explore the interactions of DElncRNAs, DEcircRNAs, and DEmRNAs in the high- and low-fertility groups, we intersected the predicted cis-targeted regulatory lncRNAs with the differential mRNAs of pituitary samples to predict genes that might be cis-targeted by lncRNAs. The predicted cis-targeted regulatory relationships showed that a total of 25 differentially expressed mRNAs were screened, and 51 lncRNAs were involved in gene regulation through cis-targeted regulatory effects (Supplementary Table S8; Figure 5A). The predicted circRNA–mRNA regulatory relationships revealed that a total of 31 differentially expressed mRNAs were screened, and 90 circRNAs were involved in gene regulation (Supplementary Table S9; Figure 5B).

### 3.5 Protein interaction network of differentially expressed mRNAs

**FIGURE 6:**
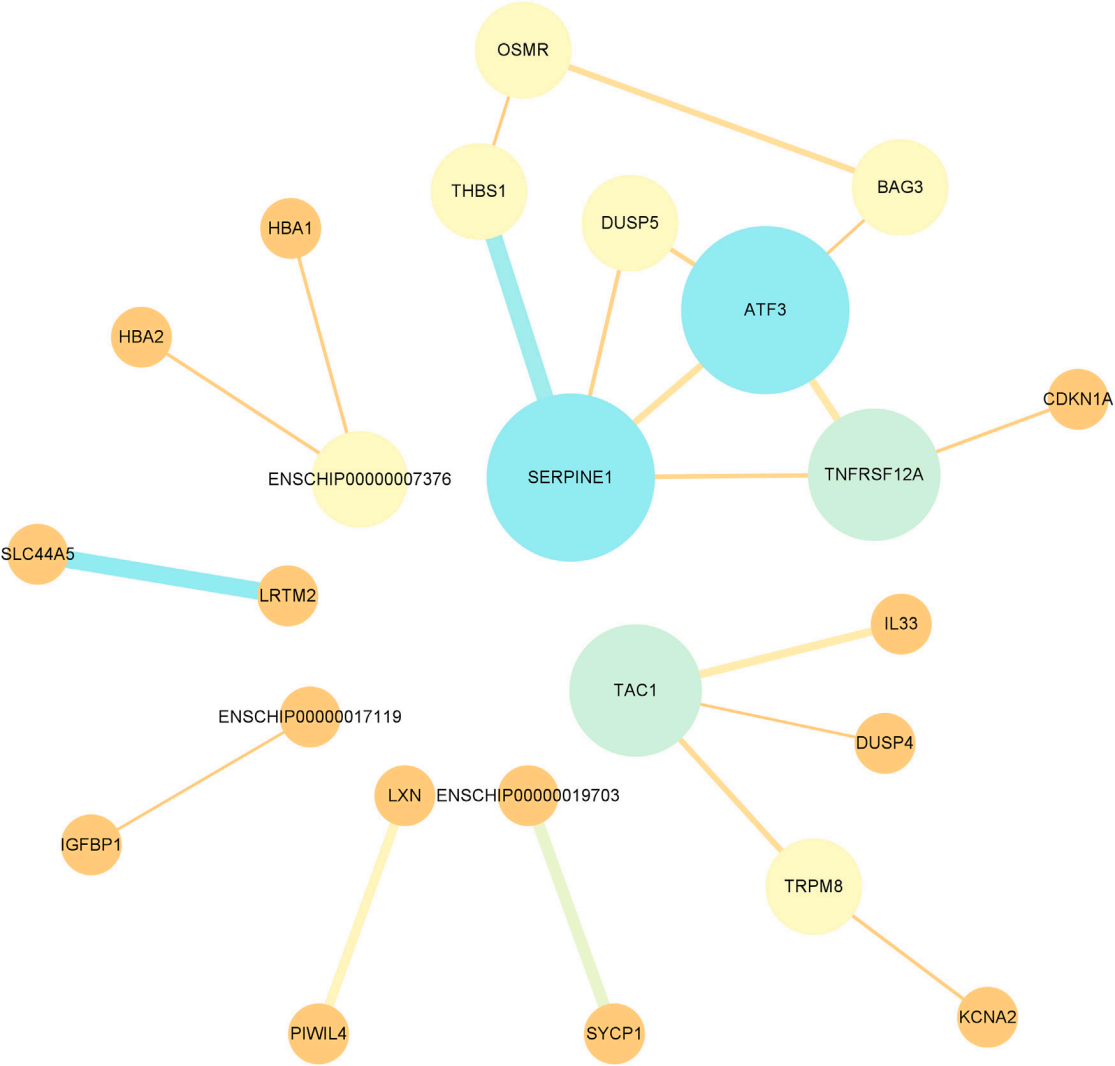
Protein–protein interaction network of DE mRNAs. Nodes in the network represent proteins, the width of the line in the node indicates the interaction between two proteins, and a wide line indicates a strong interaction.

Protein interaction networks were constructed using the STRING database to analyze the pairs of differentially expressed mRNAs and explore the significance of protein interrelationships in the pituitary in the low- and high-fertility groups. The differential mRNAs were enriched to a total of 84 proteins, and after hiding the proteins that did not contain interactions among them, 24 had interactions. Among them, SERPINE1 (degree = 4), ATF3 (degree = 4), TAC1 (degree = 3), and TNFRSF12A (degree = 3) were strongly correlated with the other proteins that may play an important role in pituitary regulation of fertility (Supplementary Table S10; Figure 6).

### 3.6 Sequencing data validation

**FIGURE 7:**
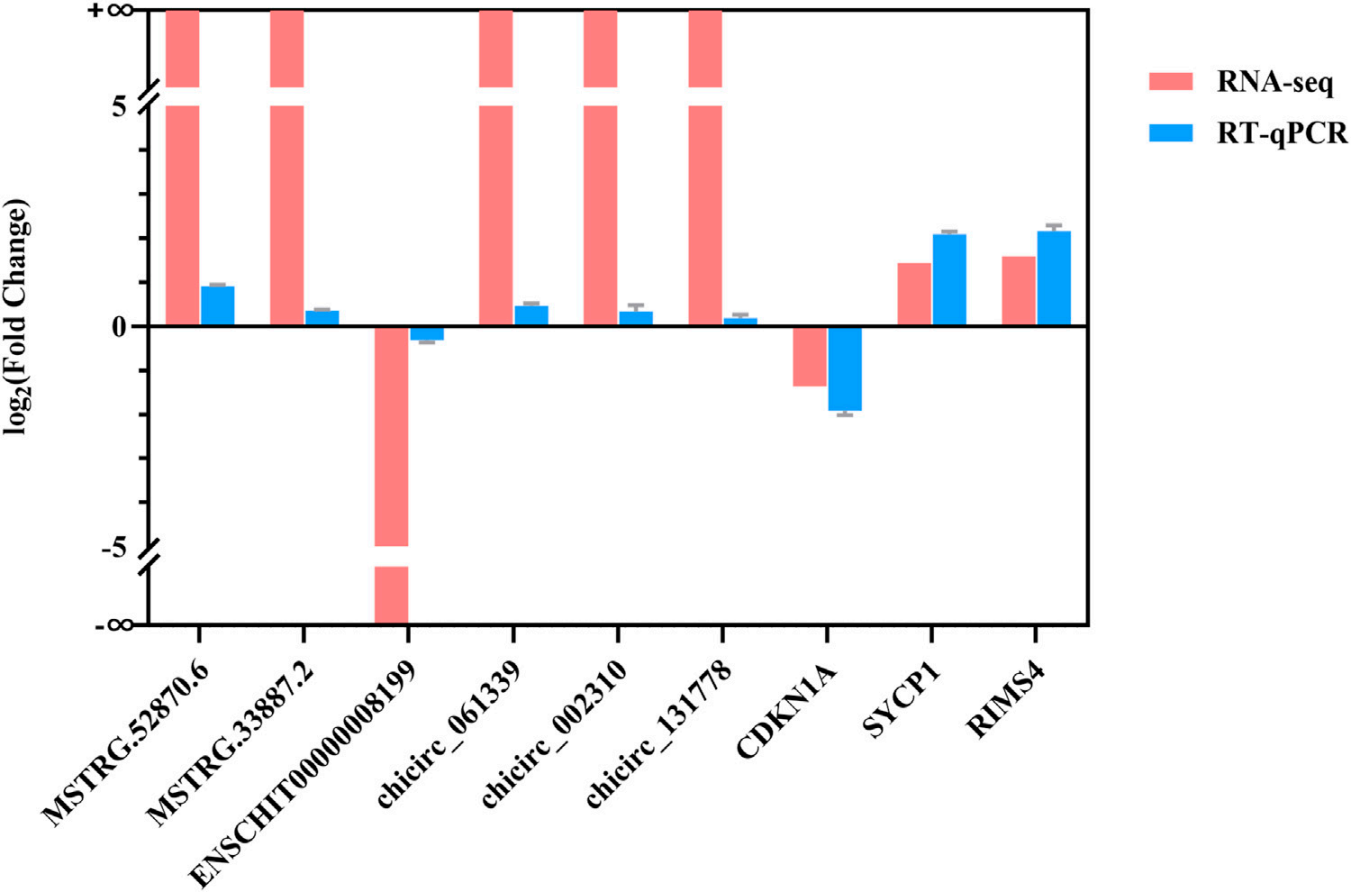
RT-qPCR verification of RNA-seq results. The X-axis represents DE lncRNAs, DE circRNAs, and DE mRNAs, and the Y-axis represents the log2 (fold change) of RT-qPCR and RNA-Seq.

The nine differentially expressed lncRNAs, circRNAs, and mRNAs were validated by RT-qPCR (Figure 7) to validate the RNA-seq results. The results showed that the expression levels of the two differed, but their trends were consistent, indicating that the sequencing results were credible.

## 4 Discussion

Goats have good adaptability to harsh environments, so they are widely distributed worldwide. Goats are one of the important sources of meat and milk, and they have high economic value in many regions (Fatet et al., 2011; Pulina et al., 2018; Muriuki et al., 2019). The pituitary gland can influence the ovarian function and follicular development by synthesizing follicle-stimulating hormone (FSH) and luteinizing hormone (LH; Sheng et al., 2021). Therefore, the pituitary gland’s influence on goat fertility can be studied by examining the pituitary function and the levels of the hormones that it regulates to obtain insights into the effects of the pituitary gland on goat fertility. The ovary, as a reproductive organ in female animals, also plays an essential role in the reproductive system, but due to the complexity of the internal structure of the ovary, determining the site that plays a role in regulating fertility in goats is difficult, and it may be affected by numerous interfering factors. Therefore, we chose to study the pituitary gland rather than the ovary to investigate high-fertility traits in Leizhou goats. Increasing evidence has shown that noncoding RNA and mRNA play an important role in goat reproduction (Ling et al., 2017; Li Y. et al., 2021). However, studies on noncoding RNA and mRNA in goat pituitary are relatively limited. Therefore, screening for genes associated with hormonal regulation is necessary to promote follicle development, increase ovulation numbers and litter size, and enhance goat fertility.

LncRNA is a new type of regulatory RNA, which is a ncRNA with a length of more than 200 bp. LncRNA is an important component of ncRNA (Shi et al., 2022), and it plays an essential role in goat ovulation and kidding mainly by regulating transcription and post-transcription (Lian et al., 2020). LncRNA also plays an important role in the regulation of the fertility network. The main ways through which lncRNA affects goat ovulation are cis (Ma et al., 2018), trans, and competitive endogenous RNA. lncRNA can also influence goat ovulation by affecting hormone secretion. In the present study, 11 differentially expressed lncRNAs were identified in the cis-regulatory effects in the pituitary gland of the low- and high-fertility groups. Enrichment analysis of the target genes of the 11 differentially expressed lncRNAs revealed that TAC1, DUSP5, and MKRN3 were enriched to pathways associated with hormonal regulation of the gonadal axis. Substance P (SP) and neurokinin A (NKA) are tachykinins encoded by the TAC1 gene. SP and NKA can regulate the secretion of GnRH and LH. They play a neuromodulatory role in reproductive processes in vertebrate animals (Arisawa et al., 1989; Ogawa et al., 2021). SP signaling in TAC1 neurons is linked to kisspeptin signaling at the level of GnRH neurons to regulate fertility in male mice (Maguire et al., 2017), but the regulation of GnRH by SP may be the opposite in different species; for example, in crested newt, SP has been shown to downregulate GnRH and attenuate the pituitary secretion of LH (Gobbetti et al., 2000). The differential spliceosome of TAC1 can produce two isoforms, which encode tachykinins that regulate prolactin (PRL) release (Pan et al., 2021). Increased PRL interferes with the hypothalamus’ secretion of GnRH, leading to decreased pituitary secretion of LH and FSH (Matuszewska et al., 2023). In our study, we found that lncRNAs (ENSCHIT00000010293, ENSCHIT00000010304, ENSCHIT00000010306, ENSCHIT00000010290, and ENSCHIT00000010298) may act through cis-targeted regulation to regulate TAC1. Therefore, the downregulation of ENSCHIT00000010293, ENSCHIT00000010304, and ENSCHIT00000010306 and the upregulation of ENSCHIT00000010290 and ENSCHIT00000010298 may cause the downregulation of TAC1, which may increase the secretion of GnRH and LH and decrease the secretion of PRL, thus enhancing the fertility of Leizhou goats. DUSP5 is a bispecific phosphatase 5 (Ni et al., 2023), and a study showed a 3.6-fold increase in the mRNA level of DUSP5 after 1 h of treatment with GnRH in hypothalamic neurons GT1-7 cells (Higa et al., 2018). Precise regulation of the synthesis and secretion of LH and FSH secreted in the pituitary gland is essential for the reproductive function of goats, and because GnRH is the most important factor in regulating the synthesis and secretion of LH and FSH, DUSP5 may play an important role in the regulation of LH and FSH secretion. In our study, we found that lncRNAs (ENSCHIT00000006769 and ENSCHIT00000006767) may regulate DUSP5 through cis-regulatory effects. MKRN3 exerts a repressive effect on GnRH secretion, and MKRN3 inhibits GnRH secretion by suppressing the transcription of KISS1 and TAC3 in hypothalamic kiss1 neurons (Abreu et al., 2021; Liu et al., 2023). According to our sequencing results, lncRNA (ENSCHIT00000006921) cis-targeted and regulated MKRN3. Therefore, the upregulation of ENSCHIT00000006921 may lead to the downregulation of MKRN3, thereby attenuating its inhibitory effect on GnRH secretion as a means to regulate the secretion of LH and FSH by the pituitary gland of Leizhou goats to enhance the fecundity of these goats. RORA is enriched to the pathway associated with steroid secretion, and it is a transcriptional regulator of the steroid hormone receptor superfamily, which transcribes steroid-related genes to regulate estrogen synthesis (Yang et al., 2022). RORA is a potential target of estrogen and androgen receptors, and it can be involved in neurodevelopment, metabolism, and immunity. Some studies have shown that mutations in RORA cause problems with fertility in sheep (Yiğit et al., 2023). RORA regulates estrogen and androgen synthesis by regulating the transcription of CYP19A1 and HSD17B10 (Sarachana and Hu, 2013). According to our sequencing results, lncRNA (ENSCHIT00000001330) cis-targeted RORA. Upregulation of ENSCHIT0000000133 leads to upregulation of RORA, which promotes the synthesis of estrogen and androgen, thereby enhancing the fertility of Leizhou goats. Therefore, these DE lncRNAs may play an important role in regulating female reproductive performance.

CircRNAs are a special class of noncoding RNAs (ncRNAs) that, unlike linear RNAs, are covalently closed-loop structures produced by reverse splicing of pre-mRNAs (Xiong et al., 2022). CircRNAs are highly stable and conserved across species because of their cyclization specificity (Zhang et al., 2019), and circRNAs can interact with RNA-binding proteins to regulate gene expression (Wang et al., 2020). Therefore, studying the function of circRNAs is worthwhile. In this study, six differentially expressed circRNAs were found to regulate the pituitary gland in the low- and high-fertility groups. Enrichment analysis of the host genes of these six differentially expressed circRNAs showed that tumor suppressor M (OSMR) was enriched in pathways related to hormone secretion. The mRNA abundance of OSMR increased after GnRH injection in ovulatory cattle, and the abundance of the X-linked inhibitor of apoptosis protein (XIAP) decreased in FSH-treated granulosa cells induced by OSM treatment. XIAP can inhibit granulosa cell apoptosis (Martins et al., 2019), so OSMR may regulate granulosa cell apoptosis under the action of hormones, such as GnRH and FSH. According to our sequencing results, OSMR expression was higher in the low-fertility group than in the high-fertility group, so OSMR may also regulate apoptosis in cells that regulate the secretion of hormones by the pituitary gland. Chicirc_029285, chicirc_026618, and chicirc_129655 jointly regulated the expression of OSMR. Analysis of the regulatory network showed that GABBR2 was related to gonadotropin secretion (Walker et al., 2012), so GABBR2 may affect the regulation of LH and FSH secretion. According to our sequencing results, chicirc_018248, chicirc_122554, chicirc_087101, and chicirc_078945 co-regulated GABBR2. Therefore, these DE circRNAs may play an important role in regulating female reproductive performance.

A total of 20,844 mRNAs were identified in the pituitary tissues of Leizhou goats in the low- and high-fecundity groups. Among them, 84 DE mRNAs were significantly different. GO and KEGG enrichment analyses of the 84 genes showed that GABBR2, SYCP1, HNF4A, CBLN1, and CDKN1A were enriched in pathways related to the regulation of gonadal axis hormones and the regulation of reproductive performance. GABBR2 belongs to the G protein-coupled receptor (GPCR) family and the GABA-B receptor subfamily. Mouse GPCR binds to relaxin-3 to increase the plasma levels of LH, which is important for follicular development because it promotes the production of androgens by follicular membranous cells; then, the follicular granulosa cells convert the androgens produced by the membranous cells into estrogens in response to the stimulation of the hormone FSH to promote follicular development. The peaks of LH are essential for ovulation in female mammals. In addition, GABBR2 is highly expressed in tilapia FSH cells and may be involved in the regulation of FSH secretion. In our sequencing results, GABBR2 was upregulated by 3.07-fold in the high-fertility group, and it may affect follicular development and ovulation in goats by influencing the secretion of LH and FSH, resulting in differences in goat reproductive performance (McGowan et al., 2008; Bathgate et al., 2013; Hollander-Cohen et al., 2021). SYPC1 plays an important role in meiosis. SYCP1 is required for the formation of crossovers in prophase meiosis, and it plays a role in regulating oocyte development (Tao et al., 2021). HNF4A has an active role in ovarian follicle differentiation and a positive role in ovarian follicular differentiation. FSH may optimize lipid metabolism in follicular tissue through the expression of HNF4A, thereby maintaining normal ovarian function (Khan et al., 2016). CBLN1 is involved in the proliferation and differentiation of nerve cells (Chen et al., 2021). CBLN1 transcription is affected by local changes in steroids (Whiley et al., 2020), so CBLN1 may play an important role in regulating goat fecundity. In our sequencing results, SYCP1, HNF4, and CBLN1 were upregulated by 2.71-fold, 20.65-fold, and 2.52-fold, respectively, in the high-fertility group. The role they plays in the pituitary gland has not been reported yet, and we hypothesize that it may regulate the hypothalamic–pituitary–gonadal axis by modulating the activities of some pituitary neurons, thus affecting the secretion of related reproductive hormones and influencing the fertility of Leizhou goats. Studies have shown that the expression of CDKN1A varies at different follicular stages, and the expression of CDKN1A in the granulosa cells of dominant follicles is lower than that in the granulosa cells of preovulatory follicles (Jiang et al., 2015). CDKN1A can reduce the proliferation of granulosa cells, but it can promote the differentiation of granulosa cells (Wissing et al., 2014). Elevated CDKN1A expression may lead to pituitary hypoplasia (Gergics et al., 2015). CDKN1A was downregulated by 0.39-fold in the high-fecundity group in our sequencing data, so CDKN1A may affect pituitary development and thus reduce fecundity in Leizhou goats. The PPI network showed that SERPINE1, TAC1, and ATF3 were strongly correlated with other proteins, and they may play an important role in the pituitary regulation of fecundity. SERPINE1 is an inhibitor of plasminogen activation 1, and it is a tissue fibrinolytic activator and major inhibitor of urokinase (Chen et al., 2022). Studies have shown that SERPINE1 is expressed in preovulatory follicles in a tissue-specific manner and can inhibit FSH-induced PGE2 production by porcine cumulus granulosa cells (Blaha et al., 2019). Research has also provided evidence of an association between estrogen agonists and SERPINE1 levels (Ghaderian et al., 2021). SERPINE1 can be used as a biomarker for the prediction of pituitary dysfunction in patients with traumatic brain injury (Frendl et al., 2017). Therefore, the mechanism for the 0.07-fold downregulation of SERPINE1 in the high-fertility group may be that SERPINE1 attenuates the regulation of the levels of other hormones that regulate the fertility of Leizhou goats by affecting the inhibitory effect of FSH secreted by the pituitary gland. TAC1 is commonly expressed in interneurons and involved in the regulation of local circuits in the brain nucleus (Burbach, 2016). The regulation of pituitary hormone secretion by TCA1 has been discussed above. ATF3 selectively stimulates FSHB expression during *in vitro* experiments, but FSH synthesis is not impaired in ATF3 knockout mice under *in vivo* conditions, so ATF3 may play a secondary role in the GnRH induction of FSHB transcription *in vivo* (Alonso et al., 2023). Moreover, ATF3 acts as a transcriptional repressor in gonadotropins (Mayer et al., 2008), and this study’s sequencing showed that ATF3 was downregulated by 0.37-fold in the high-fertility group. Thus, the reduction of ATF3 in the high-fertility group may have attenuated the repressive effect on gonadotropins. Although the roles of some central proteins in the PPI network in the pituitary have not been reported, we hypothesize that these central proteins may affect the secretion of reproductive hormones by regulating the hypothalamic–pituitary–gonadal axis. The effects of these central proteins on the number of lambs produced by Leizhou goats deserve further investigation.

## 5 Conclusion

In this study, the pituitary glands of Leizhou goats in low- and high-fecundity groups were utilized as the research objects. LncRNA, circRNA, and mRNA in the pituitary glands of Leizhou goats in the low- and high-fecundity groups were identified by RNA sequencing technology, and 11 differentially expressed lncRNAs were obtained after differential expression analysis. Six differentially expressed circRNAs and 84 differentially expressed mRNAs were identified. The target gene prediction and target relationship were further analyzed to explore the molecular mechanism of pituitary regulation of fecundity, and lncRNA–mRNA homeopathy targeting the regulatory network and the circRNA–mRNA regulatory network was established. DE lncRNAs, DE circRNAs, and DE mRNAs that regulated GnRH, LH, and FSH secretion and reproductive traits were screened out. The results can help reveal the molecular mechanisms of pituitary regulation of fertility in Leizhou goats and serve as a theoretical basis for investigating the fecundity of Leizhou goats.

## Data Availability

The data presented in the study are deposited in the NCBI repository: https://www.ncbi.nlm.nih.gov/, accession number PRJNA1043736.
